# Data-Driven Polymer Classification Using BiGRU and Hybrid Metaheuristic Optimization Algorithms

**DOI:** 10.3390/polym17141894

**Published:** 2025-07-09

**Authors:** Mohammad Anwar Parvez, Ibrahim M. Mehedi

**Affiliations:** 1 Department of Chemical Engineering, College of Engineering, King Faisal University, Al-Ahsa 31982, Saudi Arabia; 2 School of Robotics, XJTLU Entrepreneur College (Taicang), Xi’an Jiaotong-Liverpool University, No. 111 Taicang Ave., Taicang, Suzhou 215400, China

**Keywords:** deep learning (DL), data-driven polymer classification, zebra optimization algorithm, feature selection, data normalization

## Abstract

Polymers characterize a different and important class of materials through various industries, all with unique functional properties and structural attributes. Conventional models of polymer classification depend greatly on labor-intensive methods liable to human error and subjectivity. Hence, a continually growing requirement for new polymers with greater properties is a deep understanding and exploration of the chemical space. Hence, data-driven methods for polymers are developing and able to deal with unique challenges originating from the outstanding physical and chemical range of polymers at smaller and larger scales. Recently, Deep Learning (DL) models have considerably transformed material science by allowing for the automatic study and classification of composite polymers. In this paper, a novel optimization algorithm with a DL-Based Neural Networks for Data-Driven Polymer Classification (OADLNN-DDPC) model is proposed. The main intention of the OADLNN-DDPC model is to improve the classification model for data-driven polymers using state-of-the-art optimization algorithms. The data normalization stage is initially executed via Z-score normalization to convert input data into a beneficial format. In addition, the proposed OADLNN-DDPC model implements the bald eagle search (BES) model for feature selection to detect and retain the most appropriate features. For the polymer classification process, the bidirectional gated recurrent unit (BiGRU) technique is employed. Lastly, the zebra optimizer algorithm (ZOA) is implemented for the tuning process. Extensive experiments conducted on a polymers dataset with 19,500 records and 2048 features demonstrated that OADLNN-DDPC achieves an accuracy of 98.58%, outperforming existing models, such as LSTM (83.37%), PLS-DA (88.18%), and K-NN (98.36%). The simulation process of the OADLNN-DDPC model is performed under the polymer classification dataset. The experimental analysis specified that the OADLNN-DDPC model demonstrated improvement over another existing model.

## 1. Introduction

Polymers play a crucial role in various industries, including medicine, electronics, and energy. However, their classification remains a challenge due to their diverse chemical structures, physical properties, and complex molecular interactions. Traditional classification approaches rely on manual feature extraction and rule-based methods, which are time-consuming and prone to subjectivity. Furthermore, polymer characterization requires advanced predictive modeling to accelerate the discovery of new materials. Polymers are highly co-related many-body methods with intricate frameworks and dynamics spanning a broad array of time scales and lengths. Their relaxation processes comprise complex phenomena, like gelation, vitrification, semi-crystallization, and jamming, that are highly process-dependent and still lack comprehensive theoretical frameworks [1]. These phenomena are strongly affected by the chemical knowledge of a polymer’s building blocks. The optimum utilization of polymers in cutting-edge technologies like medicine, electronics, and energy gadgets requires a deeper understanding of the connections between molecular chemistry and materials processing and the determination of approaches for their quick and rational design [2]. Developments in theoretical, experimental, and computational polymer investigations and their close incorporation may enhance the existing knowledge of polymers and speed up their design by producing a huge amount of data [3].

The intentions of innovative polymer materials are inspected at three levels of enlargement. During the initial phase, researchers depend on experimentally driven trial-and-error methods to originate materials [4]. This begins with the determination of an issue or hypothesis, followed by testing with a projected solution, and eventually learning from failure for the subsequent iteration [5]. Nevertheless, the complicated approach in this phase has restrictions, like unexpected preparations and discoveries from standard chemical compounds built in nature; therefore, their abilities in the subsequent novel formulations are limited [6]. Additionally, they are highly labor-intensive, cost-intensive, and time-consuming. In another phase, investigators utilize higher-throughput investigations or virtual screening to establish the related assets of huge targets, and they select the finest ones for advanced optimization [7]. During the third phase, an investigation paradigm deals with the material–property concerns in an inverted manner. That method determines the procedure to establish the desired properties-to-appropriate materials relationship. This is referred to as inverse design, or the structure-to-property process framework [8]. With progress in DL and Machine Learning (ML) and a novel inspection model, effective gadgets to navigate the intention space have been developed. Artificial Intelligence (AI) is utilized to forecast polymer assets and to find a mapping-function-relevant framework for the property of choice [9]. Deep generative techniques are used to search to learn the essential probability distribution of frameworks and their equivalent assets to link them using a non-linear method. The DL models are also used as the suggested method for generating a hypothesis about the test conditions, which makes it possible to produce polymers [10].

In this study, a novel optimization algorithm with DL-Based Neural Networks for Data-Driven Polymer Classification (OADLNN-DDPC) model is proposed. The main intention of the OADLNN-DDPC model is to improve the classification model for data-driven polymers using state-of-the-art optimization algorithms. The data normalization stage is initially executed via Z-score normalization to convert the input data into a beneficial format. In addition, the proposed OADLNN-DDPC model implements the bald eagle search (BES) model for feature selection to detect and retain the most relevant features. For the polymer classification process, the bidirectional gated recurrent unit (BiGRU) technique is employed. Finally, the zebra optimization algorithm (ZOA) is implemented for the tuning process. The simulation process of the OADLNN-DDPC model is performed under the Polymers classification dataset. Despite their high accuracy, the DL models used in this study, such as BiGRU, may still suffer from overfitting, particularly on smaller datasets or when the model’s complexity exceeds the available data size. While the OADLNN-DDPC model shows a high performance, the optimization algorithms, such as BES and ZOA, add an additional computational overhead, which may affect the real-time applications or when working with larger datasets. With advancements in Deep Learning (DL) and Machine Learning (ML), the automated classification of polymers has gained significant attention. Recent studies have explored Convolutional Neural Networks (CNNs), Recurrent Neural Networks (RNNs), and transformer-based architectures for material classification. However, these models often suffer from overfitting, high computational complexity, and suboptimal feature selection. To address these limitations, this study proposes OADLNN-DDPC, an optimized DL framework leveraging the following:

1. Z-score normalization to standardize input data distribution;
2. Bald Eagle Search (BES) optimization for selecting the most relevant polymer features;
3. Bidirectional Gated Recurrent Unit (BiGRU) for learning complex polymer representations;
4. Zebra Optimization Algorithm (ZOA) for hyperparameter tuning, optimizing BiGRU performance.

By integrating state-of-the-art optimization algorithms with DL, OADLNN-DDPC enhances both the classification accuracy and computational efficiency. Section 2 reviews the existing literature on AI-driven polymer classification, discussing various DL architectures, such as CNN, RNN, and transformer models, that have been employed in previous studies. Section 3 explains the pre-processing, feature selection, classification method. Section 4 presents the experimental setup and the results of the OADLNN-DDPC model when tested on the Polymer classification dataset. It includes detailed performance metrics, such as accuracy, precision, recall, F1-score, and execution time under various experimental conditions (70:30 training and testing). The section compares these results with existing methods, demonstrating the superiority of the OADLNN-DDPC model in terms of the classification performance and efficiency. Finally, Section 5 concludes the research.

## 2. Related Works

Several studies have explored AI-driven polymer classification. Velazquez et al. [11] introduced a robotic three-dimensional printer particularly intended for UV-curable thermosets, whose printing settings could be favored by using a predictive modeling technique. Software packages are advanced to allow for the interaction among the control systems, the robotic arm, and the extruder head to control the printing process. A predictive method employing both CNN and FNN is developed for calculating the sizes of upcoming prints depending on the process parameters. Cui and Wang [12] developed an innovative method for assessing the consistency among asphalt binders and waste polymers employing cutting-edge molecular representation techniques. The waste polymer’s solubility parameters are forecast by utilizing a geometry-enhanced graph neural network (GeoGNN) and conventional ML approaches, respectively. The compatibility index is estimated to depend on the complete variance among the solubility parameters of asphalt and polymers. Shi et al. [13] introduce optical employment for quick monitoring with minimal digital post-processing, utilizing optical data processing. This study incorporates a diffractive-enhanced optical neural networking with a stacked liquid crystal electrical control design, merging the parallel processing capability. Over a single-time effort training, the system allows for precise forecasting of the preferred liquid crystal molecules that are arranged over numerical blind testing.

In [14], a Bi-Stage Optimize DNN (BSO-DNN) with a TL approach that is optimized for diverse resources effectively enhances the precision of modeling and utilizing data. This model substantially improves the sturdiness of the method, the forecast values are closest to the real-sample dispersions, and the precise dispersions give dependable patterns and admissible values for the advanced utilization of the resources. Dai et al. [15] developed three NN techniques comprising LSTM, DNN, and RNN to predict the cohesive zone parameter, as the nickel content in modified carbon nanotubes varied, thus simplifying the challenge of obtaining CZM parameters for nanoparticle-reinforced adhesives. These three types of networks are skilled and depend on the enhanced hyperparameters attained from the Bayesian hyperparameter-tuning process.

Ranjbar et al. [16] developed a detailed examination, utilizing RGB images for the DL-based classification by form of resin. This database included four generally utilized plastic kinds of project structure: PVC, PS, HDPE, and ABS. Utilizing TL with pre-trained models on ImageNet, the utmost precision method designed for this classification job is presented in this study. In [17], the data-driven models are applied for improving machine-recognizable polymer representations and to manage the huge physical and chemical instability of polymers at diverse scales. Progressive generative AI approaches are utilized for the backward design of polymers with particular assets, making them suitable for several applications. Liu [18] developed a multi-modal DL framework integrating spectral imaging and molecular fingerprint data, achieving high classification accuracy for polymer analysis. Xu et al. [19] introduced a transformer-based model for polymer sequence classification, significantly reducing the training time while maintaining high accuracy. Xu et al. [20] proposed an unsupervised deep clustering approach for polymer categorization, which improved the generalization across various polymer types. Metha et al. [21] designed a federated learning framework for polymer classification, ensuring a privacy-preserving data analysis across multiple laboratories. Ballard et al. [22] applied reinforcement learning techniques to optimize polymer classification pipelines, dynamically adjusting feature selection for enhanced performance. Unlike previous studies, OADLNN-DDPC combines multiple optimization techniques (BES and ZOA) with DL to improve the feature selection, classification accuracy, and computational efficiency. The main contributions of the proposed methods are as follows:

Novel Methodology: This study proposes the OADLNN-DDPC model, which integrates multiple state-of-the-art optimization techniques (BES and ZOA) with DL methods for the polymer classification, marking a significant contribution to the field.

Enhanced Feature Selection: Utilizing Bald Eagle Search (BES) for the feature selection guarantees that only the most pertinent features are employed in the classification, leading to increased model efficiency and accuracy.

Enhanced Classification Accuracy: The proposed model achieves an accuracy of 98.58%, significantly outperforming existing models such as LSTM, PLS-DA, and K-NN.

Reduced Execution Time: The OADLNN-DDPC model not only outperforms existing models in terms of accuracy but also achieves the best execution time (10 min), making it practical for real-world applications.

Robust Hyperparameter Tuning: By implementing a Zebra Optimization Algorithm (ZOA) for hyperparameter tuning, the model optimizes the BiGRU performance, ensuring both a high classification accuracy and a high computational efficiency [23,24].

## 3. Proposed Methodology

**Figure 1:**
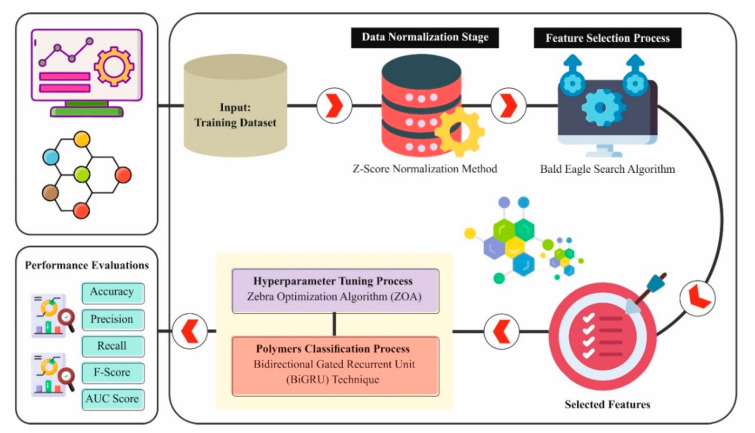
Overall flow of the OADLNN-DDPC model.

The BES algorithm was selected for its proven capability to maintain a balance between local exploitation and global exploration, which is essential in high-dimensional feature spaces. BiGRU was chosen over traditional RNNs and LSTMs due to its reduced parameter count, faster convergence, and ability to capture contextual dependencies from past and future data. ZOA was used for hyperparameter tuning because of its ability to avoid local minima, dynamic population diversity, and competitive performance compared to other metaheuristics like PSO and GA. In this study, a novel OADLNN-DDPC method is presented. The main intention of the OADLNN-DDPC approach is to improve the classification model for data-driven polymers using state-of-the-art optimization algorithms. The proposed OADLNN-DDPC contains various levels, such as data normalization, feature selection, classification, and hyperparameter tuning, to attain this. Figure 1 depicts the complete workflow of the OADLNN-DDPC model [25,26].

### 3.1. Pre-Processing

Initially, the Z-score normalization, as standardization, is accomplished for converting input data into a beneficial format, which converts data by subtracting the mean of all features and dividing them by their standard deviation. For the data-driven polymer classification, this model guarantees that every feature has a standard deviation of 1 and a mean of 0, making them similar despite their novel unit or scales [18]. This was mainly useful after addressing features that are associated with changing distributions, as it aids in alleviating the influence of features with large numeric ranges. This approach also improves the performance and stability of classification techniques, like neural networks or SVMs, by ensuring that the optimization process treats each feature appropriately. Nevertheless, it is aware of outliers; they can expressively influence the standard deviation and mean. Regardless, it is normally utilized once, preparing the polymer datasets for ML tasks. Prior to the Z-score normalization, missing values were imputed using median substitution to minimize the bias. Outliers were capped at the 1st and 99th percentiles using an interquartile range (IQR)-based method. This preprocessing step ensured that extreme values did not unduly influence the model training. Post-normalization, distributions were visualized to confirm consistent scaling [27].

### 3.2. Feature Selection Process

In addition, the proposed OADLNN-DDPC models implement the BES model for the feature selection to detect and retain the most relevant features from the data. The bald eagle utilizes an advanced hunting tactic, particularly after hunting salmonid prey inside marine environments [19]. Utilizing a systematic method, the eagle started its hunting expedition by accurately measuring wide-ranging water bodies, using its broader vision related to advanced optical instruments. This primary reconnaissance stage is essential, allowing the bird to distinguish the dynamic movements and attentions of salmon populations. The obtained insights into prey abundance develop the foundation for the following searching phases, highlighting the bald eagle’s precision and adaptability in optimizing the searching success.

Regarding the searching behaviors shown by bald eagles, we newly presented the BES optimizer, an advanced swarm-based model stimulated by the extraordinary searching approaches of this avian predator. Imitating the bald eagle’s three-stage pursuit habit, this original optimizer originates with a space selection stage, tactically identifying areas with a greater probability of harboring the best solutions. Then, the searching stage is explained as the model dynamically discovers the selected region, reflecting the eagle’s in-depth scanning for prey. The termination occurs in the swoop stage, in which the optimizer, directed by previous insights, accurately enhances the best solution with effectiveness. By manufacturing the complex behaviors of nature’s predators, the BES supports a swarm-based model, providing an optimistic chance for improved computation problem-solving across different fields. During the underlying BES, bald eagles search for food over three phases: the swooping, the search, and the select stages. The mathematical representations are defined below.

#### 3.2.1. Initialization

Like another model, the BES searches for optimum solutions by making primary random populations inside the candidate solution’s boundaries. Provided that there are N populations, all containing dim decision variables, the arbitrary population is made based on the succeeding Equation (1):

(1) $$P_{i}^{j}=l_{b}+rand\times \left(u_{b}-l_{b}\right),\left\{\begin{array}{c} i=1,2,\ldots ,N.\\ j=1,2,\ldots ,dim. \end{array}\right.$$

where rand characterizes a randomly generated number among (0, 1), $u_{b}$ and $l_{b}$ specify the upper and lower limits, and $P_{i}^{j}$ signifies the location of j th dimension in the ith population.

#### 3.2.2. Selecting Phase

During this stage, all bald eagles choose the region according to the optimal location (for example, food position) and average location that can be expressed as demonstrated in Equation (2):

(2) $$P_{i,new}=P_{best}+\alpha \times r_{6}\times \left(P_{mean}-P_{i}\right)$$

where $P_{best},\;\;P_{mean}$, and $P_{i}$ specify the position of the food and the average location of the present individual, correspondingly. α refers to the value between 1.5 and 2, and $r_{6}$ is a randomly generated value among (0, 1).

#### 3.2.3. Searching Phase

In this stage, the algorithm is applied to search for food near the present individual position, for example, the previously chosen position. The formulation is as shown in Equations (3) and (4):

(3) $$P_{i,new}=P_{i}+y\left(i\right)\times \left(P_{i}-P_{i+1}\right)+x\left(i\right)\times \left(P_{i}-P_{mean}\right)$$

(4) $$\left\{\begin{array}{l} x(i)=\frac{xr(i)}{max(|xr|)},y(i)=\frac{yr(i)}{max(|yr|)}\\ xr\left(i\right)=r\left(i\right)\times sin\left(\theta \left(i\right)\right),yr\left(i\right)=r\left(i\right)\times cos\left(\theta \left(i\right)\right)\\ \theta (i)=a\times \pi \times r_{7},\;r(i)=\theta (i)+R\times r_{8} \end{array}\right.$$

where a and R represent parameters confined to [5, 10] and [0.5, 2]. $r_{7}$ and $r_{8}$ denote randomly generated values in [0 1].

#### 3.2.4. Swooping Phase

In this phase, a polar equation is rooted in this method to act out the swinging behavior in pursuit. This is expressed as demonstrated in Equations (5) and (6):

(5) $$P_{i,new}=r_{9}\times P_{best}+x_{1}(i)\times \left(P_{i}-c_{1}\times P_{mean}\right)+y_{1}(i)\times \left(P_{i}-c_{2}\times P_{best}\right)$$

(6) $$\left\{\begin{array}{l} x_{1}(i)=\frac{xr(i)}{max(|xr|)},y_{1}(i)=\frac{yr(i)}{max(|yr|)}\\ xr\left(i\right)=r\left(i\right)\times sinh\left(\theta \left(i\right)\right),yr\left(i\right)=r\left(i\right)\times cosh\left(\theta \left(i\right)\right)\;\\ \theta (i)=a\times \pi \times r_{10},\;r(i)=\theta (i) \end{array}\right.$$

where $r_{9}$ and $r_{10}$ are randomly generated values in [0, 1]. $c_{1}$ and $c_{2}$ specify the swooping movement’s intensity, which falls between (1, 2). The fitness function (FF) applied in the BES methodology is to have a balance between the chosen feature amounts in the overall solutions (minimal) and the classification precision (maximal) achieved by exploiting these desired features. Equation (7) characterizes the FF to estimate outputs:

(7) $$Fitness=\alpha \gamma_{R}\left(D\right)+\beta \frac{\left|R\right|}{\left|C\right|}$$

where $\gamma_{R}(D)$ exemplifies the classification error rate. $\left|R\right|$ symbolizes the cardinality of the selected subset, and |C| specifies the total features in the data. α and β are dual parameters corresponding to the significance of subset length and classification quality. ∈ [1, 0] and β=1−α.

### 3.3. Classification Method

The BiGRU technique is deployed for the polymer classification process. GRU and LSTM are intended to resolve the gradient disappearance issue in RNNs. The GRU simplifies the network architecture by integrating the input and the forgotten gate in the LSTM [20]. This model decreases the parameter count of the model, streamlines the computation process, and makes the GRU quicker in training speed yet simpler to implement and understand. The GRU adjusts the information flow by presenting update gates and reset gates, permitting the method to determine what information must be reserved or forgotten to improve procedure sequence data.

**Figure 2:**
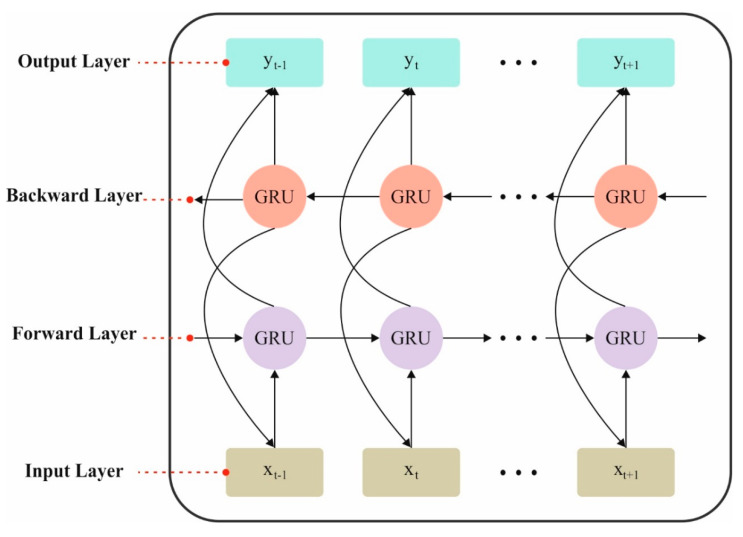
Architecture of the Bi-GRU model.

GRU networks can process information from the previous; Bi-GRU is presented to concurrently take the inspiration of upcoming details on the current input. It incorporates forward and reverse GRU, which communicate over input data, and can consider either previous or forthcoming information about the sequence. In Bi-GRU, a forward GRU addresses information from the preceding to the future; however, a reverse GRU addresses information that has never experienced the previous. Figure 2 represents the Bi-GRU framework.

In the Bi-GRU architecture, the update gate $z_{t}$ and reset the gate $r_{t}$ of the forward GRU managing the reset and update quantities of the prior hidden state and the current input, respectively. In detail, the reset gate $r_{t}$ selects what amount of information from the past hidden state $h_{t-1}$ must be reset to the primary state after computing the hidden state $h_{t}$ at the present moment. The update gate $z_{t}$ chooses the information weight retained or upgraded in the preceding HL $h_{t-1}$ after the present hidden state $h_{t}$ is upgraded. The computation process of Bi-GRU includes an amount of state transfer and gate functions, and its particular computation equation is as shown in Equations (8)–(11):

(8) $$r_{t}=\sigma \left(W_{r}\cdot \left[h_{t-1},x_{t}\right]+b_{r}\right)$$

(9) $$z_{t}=\sigma \left(W_{Z}\cdot \left[h_{t-1},x_{t}\right]+b_{Z}\right)$$

(10) $${\tilde{h}}_{t}=tanh\left(W_{h}\cdot \left[r_{t}\cdot h_{t-1},x_{t}\right]+b_{h}\right)$$

(11) $$h_{t}=\left(1-z_{t}\right)\cdot h_{t-1}+z_{t}\cdot {\tilde{h}}_{t}$$

where σ embodies the sigmoid activation function; b and W symbolize the bias vector and weight matrix, respectively.

### 3.4. Hyperparameter Tuning Model

Finally, the ZOA optimally alters the hyperparameter values of the BiGRU algorithm, resulting in a superior performance. The ZOA is a bio-inspired meta-heuristic model for optimizing difficulties [21]. It was stimulated by the zebra’s herd dynamics and social behavior in nature. Like numerous herd mammals, zebras show behaviors that support them in avoiding predators, enhancing their foraging ways, and preserving social unity. The ZOA tries to simulate the behaviors to look for optimum or near-optimum solutions in search areas. The ZOA optimized the following hyperparameters of the BiGRU model:

- Learning rate: [0.0001, 0.01];
- Number of GRU units: [32, 256];
- Batch size: [16, 128];
- Dropout rate: [0.1, 0.5];
- Number of epochs: [10, 100]: The optimization objective was to minimize the classification error while balancing the model complexity and training time.

#### 3.4.1. Herding Behavior

Zebras have a tendency to migrate in herds (groups) that assist them in avoiding predators. The model mimics this by challenging numerous candidate solutions (similar to single zebras), which pass over the searching region. The primary population of the candidate solutions (zebras) is produced arbitrarily inside the searching region. Let $x_{i}(t)$ characterize the location of the $i^{th}$ zebra within the population at the t th iteration. The primary locations $x_{i}(0)$ are typically made utilizing uniform distributions in Equation (12):

(12) $$X_{i}\left(0\right)=X_{min}+rand\;()\times \left(X_{max}-X_{min}\right)$$

Here, $x_{max}$ and $x_{min}$ represent the upper and lower boundaries of the searching region, and rand() denotes an arbitrarily generated integer among (0,1). The fitness of all zebras is assessed utilizing a pre-defined objective function $f(x_{i}(t))$, which is domain-specific and is applied to control the quality of the solution characterized by $x_{i}(t)$.

#### 3.4.2. Position Updating

The candidate solution’s positions are upgraded according to the incorporation of exploration (hunting novel regions) and exploitation (improving recent solutions). The model fine-tunes the balance amongst these dual approaches as it develops, equivalent to how zebras may modify their movement approaches regarding environmental factors. The location of all zebras is upgraded according to the social behavior of the group. The novel location $x_{j}(t+1)$ is affected by the top-performing zebras within the populace and by arbitrary features to guarantee diversity. The location upgrade is modeled by Equation (13):

(13) $$X_{i}\left(t+1\right)=X_{i}\left(t\right)+c_{1}\times rand()\times \left(X_{best}\left(t\right)-X_{i}\left(t\right)\right)+c_{2}\times (1-rand())\times (X_{avg}(t)-X_{\partial }.(t))$$

where $x_{best}(t)$ represents the position of the top-performing zebra at the tth iteration, $x_{avg}(t)$ refers to the average location of each zebra at the t th iteration, and $c_{1}$, and $c_{2}$ are constants directing the inspiration of the optimal solution and the group, correspondingly.

#### 3.4.3. Predator Avoidance and Social Interaction

Zebras interact with one another to coordinate movement and prevent danger. In the ZOA, these communications have struggled through the exchange of information among candidate solutions, allowing them to share information regarding possible locations of the searching region. The predator’s existence in nature influences zebras to continuously adjust their routes to avoid being captured. This theory has been applied in the model to present diversity in the searching procedure, averting the model from becoming trapped in local bests. To prevent local ideals and mimic predator avoidance, arbitrary disturbances are supplemented to the position of the zebra shown in Equation (14):

(14) $$X_{i}\left(t+1\right)=X_{i}\left(t+1\right)+\sigma \times rand()$$

where σ denotes a smaller constant demonstrating the disturbance intensities, and rand() denotes a randomly generated number specified from a standard distribution. After upgrading the locations, the model assesses the novel locations and chooses the top-achieving zebras to endure to the following iteration. This stage guarantees that only guaranteeing solutions affect the search procedure. The ZOA originates the FF to reach a better performance of classification. It impacts a positive number to define the superior accomplishment of the potential solutions. In this paper, the reduction in the error rate of the classifier is reflected as the FF, as provided in Equation (15):

(15) $$fitness\left(x_{i}\right)=ClassifierErrorRate\left(x_{i}\right)=\frac{no\;of\;misclassified\;samples}{Total\;no\;of\;samples}*100$$

## 4. Experimental Validation and Results

The performance evaluation of the OADLNN-DDPC model is tested under the Polymers classification dataset [22]. Polymer classification includes several key parameters, such as glass transition temperature (ranging from −70 °C to 150 °C), melting points (from 100 °C to 350 °C), viscosity (ranging from 0.01 to 1000 Pa·s), rheology (values between 0.1 and 10 Pa·s), elasticity (ranging from 1 to 10 GPa), Young’s modulus (ranging from 0.5 to 4 GPa), and resilient modulus (values between 1 and 5 GPa). These parameters provide crucial information about the physical properties and behavior of polymers. By integrating these values, the model can more efficiently capture the relationships between diverse polymer types, allowing for a more accurate classification of the three class labels, namely Plastic, Peptide, and Oligosaccharide. The inclusion of these features significantly improves the capability of the model to discern subtle distinctions and enhance overall performance in data-driven polymer classification. Table 1 specifies the dataset description, and Table 2 portrays the sample class smiles. To validate the statistical significance of OADLNN-DDPC’s performance improvements, paired t-tests were conducted, comparing it to each baseline model. Results indicated statistically significant improvements (*p* < 0.01) in accuracy and F1-score over LSTM, K-NN, and PLS-DA, confirming that the gains were not due to random variation.

**Figure 3:**
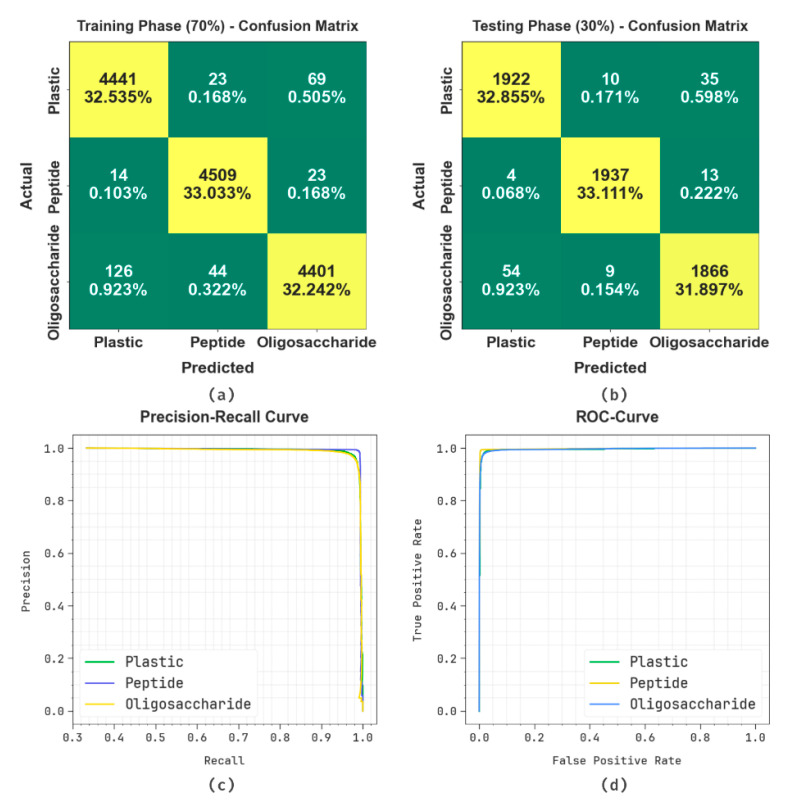
Classifier analysis of (**a**,**b**) confusion matrices and (**c**,**d**) PR and ROC curves under 70:30 of TRAPS/TESPS.

Figure 3 displays the classification performances of the OADLNN-DDPC methodology under 70:30 of TRAPS/TESPS. Figure 3a,b exemplify the confusion matrices through the precise detection and classification of all classes. Figure 3c,d exemplify the PR and ROC inspection, which indicates a superior performance over all classes. The inclusion of key polymer parameters—such as glass transition temperature, melting point, viscosity, elasticity, rheology, Young’s modulus, and resilient modulus—provides critical input to the classifier. These parameters directly influence molecular structure, thermal behavior, and mechanical performance, which are key discriminators among plastics, peptides, and oligosaccharides. Their integration improves the model’s ability to learn from structural–functional relationships.

**Figure 4:**
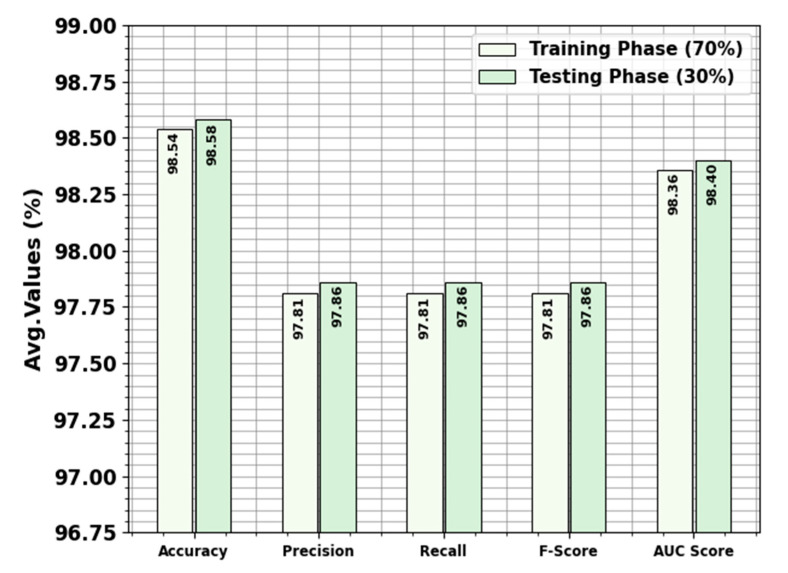
Average of the OADLNN-DDPC method under 70:30 of TRAPS/TESPS.

Table 3 and Figure 4 signify the classifier performances of the OADLNN-DDPC approach below 70:30 of TRAPS/TESPS. The outputs portrayed that the OADLNN-DDPC approach precisely recognized the samples. Using 70% TRAPS, the OADLNN-DDPC method attains an average $accu_{y}$, $prec_{n}$, $reca_{l}$, $F_{score}$, and ${AUC}_{score}$ of 98.54%, 97.81%, 97.81%, 97.81%, and 98.36%, respectively. Additionally, using 30% TESPS, the OADLNN-DDPC algorithm delivers an average $accu_{y}$, $prec_{n}$, $reca_{l}$, $F_{score}$, and ${AUC}_{score}$ of 98.58%, 97.86%, 97.86%, 97.86%, and 98.40%, respectively.

**Figure 5:**
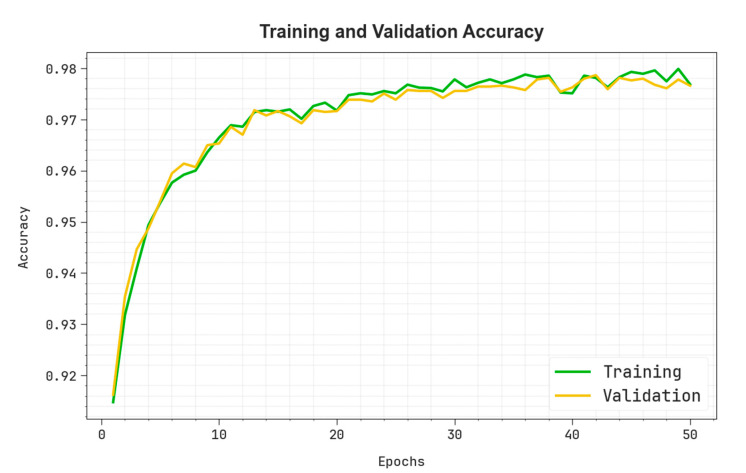
The $Accu_{y}$ curve of the OADLNN-DDPC model.

In Figure 5, the training (TRA) $accu_{y}$ and validation (VAL) $accu_{y}$ performances of the OADLNN-DDPC method are depicted. The values of $accu_{y}$ are computed through a time period of 0–50 epochs. The figure underscored that the values of TRA and VAL $accu_{y}$ exhibit growth, illustrating the capability of the OADLNN-DDPC method to enhance the performance through multiple repetitions. Moreover, the TRA and VAL $accu_{y}$ values remain close across the epochs, specifying diminished overfitting and showing the maximum performance of the OADLNN-DDPC method, which guarantees a reliable calculation on unseen samples.

**Figure 6:**
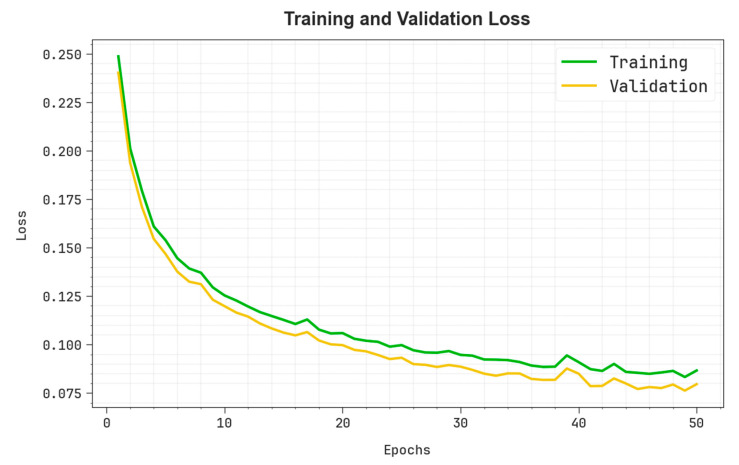
Loss curve of the OADLNN-DDPC model.

In Figure 6, the TRA loss (TRALOS) and VAL loss (VALLOS) graph of the OADLNN-DDPC algorithm is shown. The loss is computed across a time period of 0–50 epochs. It is depicted that the values demonstrate a diminishing trend, which indicates the proficiency of the OADLNN-DDPC technique in corresponding to a tradeoff between generalization and data fitting. The successive dilution in values of loss as well as securities the superior performance of the OADLNN-DDPC approach and tunes the calculation results after a while.

**Figure 7:**
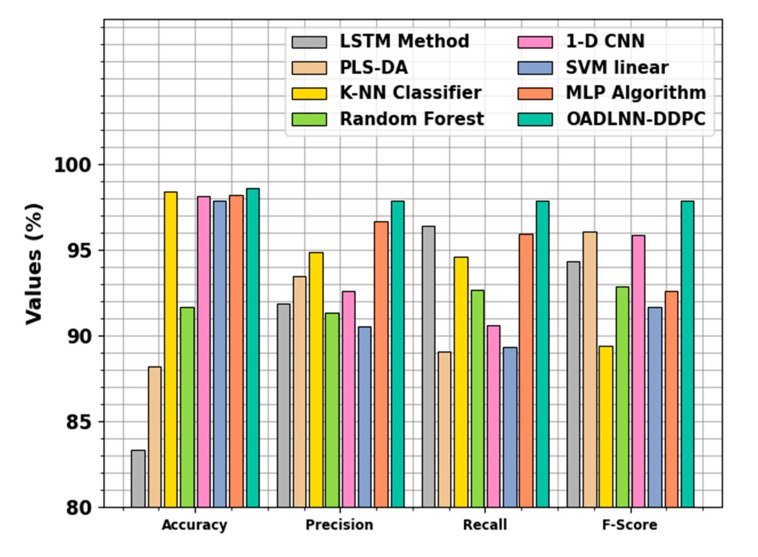
Comparison evaluation of the OADLNN-DDPC model with existing approaches.

The comparative study of the OADLNN-DDPC model with existing techniques is illustrated in Table 4 and Figure 7 [28,29]. According to $accu_{y}$, the OADLNN-DDPC system has maximum $accu_{y}$ of 98.58%, whereas the LSTM, PLS-DA, K-NN, RF, 1-D CNN, SVM linear, and MLP methodologies have minimum $accu_{y}$ values of 83.37%, 88.18%, 98.36%, 91.68%, 98.10%, 97.86%, and 98.19%, correspondingly. Furthermore, according to ${Prec}_{n}$, the OADLNN-DDPC method has an enhanced ${Prec}_{n}$ of 97.86% whereas the LSTM, PLS-DA, K-NN, RF, 1-D CNN, SVM linear, and MLP algorithms have lesser ${Prec}_{n}$ values of 91.89%, 93.49%, 94.86%, 91.35%, 92.58%, 90.56%, and 96.65%, correspondingly. Also, according to $F_{score}$, the OADLNN-DDPC system has a superior $F_{score}$ of 97.86%, whereas the LSTM, PLS-DA, K-NN, RF, 1-D CNN, SVM linear, and MLP techniques have decreased $F_{score}$ values of 94.33%, 96.08%, 89.41%, 92.84%, 95.84%, 91.64%, and 92.57%, respectively.

**Figure 8:**
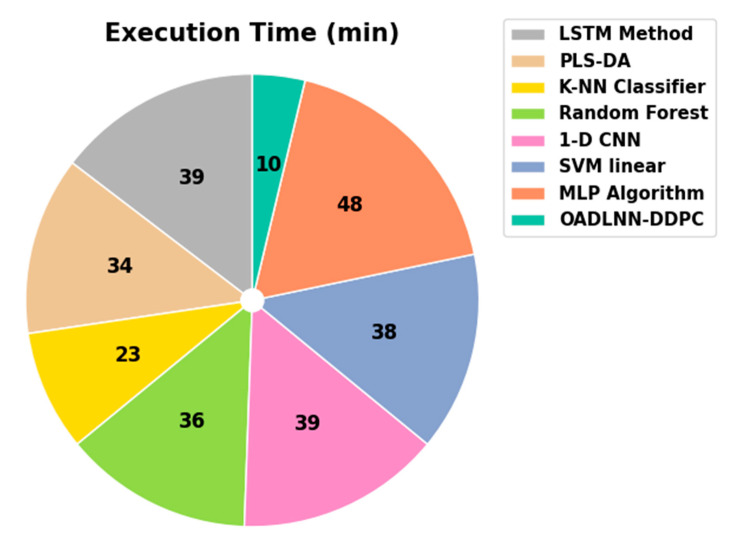
ET outcome of the OADLNN-DDPC model with present techniques.

In Table 5 and Figure 8, the comparison solutions of the OADLNN-DDPC method are detected in terms of execution time (ET). The performances imply that the OADLNN-DDPC algorithm achieves a greater performance. On ET, the OADLNN-DDPC system offers a minimal ET of 10 min, while the LSTM, PLS-DA, K-NN, RF, 1-D CNN, SVM linear, and MLP methodologies gain better ETs of 39 min, 34 min, 23 min, 36 min, 39 min, 38 min, and 48 min, respectively. For a fair comparison, all baseline models were reimplemented and trained using the same dataset, preprocessing steps, training/test split (70:30), and performance metrics. This ensured consistent evaluation across all models.

## 5. Conclusions

In this study, a novel OADLNN-DDPC methodology is presented. The aim of the OADLNN-DDPC technique is to improve the classification model for data-driven polymers using state-of-the-art optimization algorithms. The data normalization stage is initially executed by the Z-score normalization for converting the input data into a beneficial format. In addition, the proposed OADLNN-DDPC models implement the BES model for the feature selection to identify and retain the most appropriate features from the data. The BiGRU technique has been deployed for the polymer classification process. This study presents OADLNN-DDPC, a novel DL-based polymer classification model integrating optimization techniques. The proposed approach significantly outperforms existing methods in accuracy (98.58%) and execution time (10 min) while ensuring robust feature selection and model generalization. Future work will focus on extending the model to handle multi-modal polymer datasets and exploring self-supervised learning techniques for improved feature representation. Finally, the ZOA is utilized for the tuning process. The simulation process of the OADLNN-DDPC system is performed using the Polymers classification dataset. The experimental validation specified that the OADLNN-DDPC approach outperformed another existing model.

## Figures and Tables

**Table 1:** Dataset description.

Class Label	Record Numbers
Plastic	6500
Peptide	6500
Oligosaccharide	6500
Total Records	19,500

**Table 2:** Samples of class smiles.

**S.no**	Smiles	Label
1	“C(Cl)CCC(c1ccccc1)CCC(Cl)C”	Plastic
2	“CC(c1ccccc1)CCC(CC)CCCC(C)C”	Plastic
3	“OC(=O)[C@]([H])(CCSC)NC(=O)[C@]([H])(CC(C)C)NC(=O)[C@]([H])(CC(C)C)N”	Peptide
4	“OC(=O)[C@]([H])(CO)NC(=O)[C@]([H])(C1CCCN1)NC(=O)[C@]([H])([H])N”	Peptide
5	“O[C@]([C@]([H])(CO)O[C@](O)([H])[C@]([H])1O)([H])[C@]1([H])O[C@]([C@]([H])(CO)O[C@](O)([H])[C@]([H])1O)([H])[C@]1([H])O[C@@]([C@]([H])(CO)O[C@](O)([H])[C@@]([H])1O)([H])[C@]1([H])O”	Oligosaccharide
6	“O[C@]([C@@]([H])(CO)O[C@@](O)([H])[C@]([H])1O)([H])[C@@]1([H])N[C@]([C@]([H])(CO)O[C@](O)([H])[C@]([H])1O)([H])[C@]1([H])O[C@]([C@]([H])(CO)O[C@](O)([H])[C@]([H])1O)([H])[C@@]1([H])O[C@]([C@]([H])(CO)O[C@](O)([H])[C@]([H])1O)([H])[C@@]1([H])O”	Oligosaccharide

**Table 3:** Classifier analysis of OADLNN-DDPC methodology under 70:30 of TRAPS/TESPS.

Class Labels	$Accu_{y}$	$Prec_{n}$	$Reca_{l}$	$F_{score}$	$AUC_{score}$
**TRAPS (70%)**					
Plastic	98.30	96.94	97.97	97.45	98.22
Peptide	99.24	98.54	99.19	98.86	99.23
Oligosaccharide	98.08	97.95	96.28	97.11	97.63
Average	98.54	97.81	97.81	97.81	98.36
TESPS (30%)					
Plastic	98.24	97.07	97.71	97.39	98.11
Peptide	99.38	99.03	99.13	99.08	99.32
Oligosaccharide	98.10	97.49	96.73	97.11	97.75
Average	98.58	97.86	97.86	97.86	98.40

**Table 4:** Comparison evaluation of the OADLNN-DDPC model with existing approaches.

Technique	$accu_{y}$	${Prec}_{n}$	$Reca_{l}$	$F_{score}$
LSTM Method	83.37	91.89	96.42	94.33
PLS-DA	88.18	93.49	89.06	96.08
K-NN Classifier	98.36	94.86	94.60	89.41
Random Forest	91.68	91.35	92.66	92.84
1-D CNN	98.10	92.58	90.59	95.84
SVM linear	97.86	90.56	89.36	91.64
MLP Algorithm	98.19	96.65	95.91	92.57
OADLNN-DDPC	98.58	97.86	97.86	97.86

**Table 5:** ET outcome of the OADLNN-DDPC approach with existing techniques.

Technique	ET (min)
LSTM Method	39
PLS-DA	34
K-NN Classifier	23
Random Forest	36
1-D CNN	39
SVM linear	38
MLP Algorithm	48
OADLNN-DDPC	10

## Data Availability

All data generated or analyzed during this study are included in this manuscript. Additional information or supporting materials are available from the corresponding author upon reasonable request.
